# Microbes as Master Immunomodulators: Immunopathology, Cancer and Personalized Immunotherapies

**DOI:** 10.3389/fcell.2019.00362

**Published:** 2020-01-23

**Authors:** Joana R. Lérias, Georgia Paraschoudi, Eric de Sousa, João Martins, Carolina Condeço, Nuno Figueiredo, Carlos Carvalho, Ernest Dodoo, Mireia Castillo-Martin, Antonio Beltrán, Dário Ligeiro, Martin Rao, Alimuddin Zumla, Markus Maeurer

**Affiliations:** ^1^ImmunoSurgery Unit, Champalimaud Centre for the Unknown, Lisbon, Portugal; ^2^Digestive Unit, Champalimaud Centre for the Unknown, Lisbon, Portugal; ^3^University of Heidelberg, Heidelberg, Germany; ^4^Department of Pathology, Champalimaud Centre for the Unknown, Lisbon, Portugal; ^5^Lisbon Centre for Blood and Transplantation, Instituto Português do Sangue e Transplantação, Lisbon, Portugal; ^6^Division of Infection and Immunity, NIHR Biomedical Research Centre, University College London Hospitals NHS Foundation Trust, University College London, London, United Kingdom

**Keywords:** pathogens, microbiota, inflammation, neoplasia, immune responses, antibodies, cancer, immunotherapy

## Abstract

The intricate interplay between the immune system and microbes is an essential part of the physiological homeostasis in health and disease. Immunological recognition of commensal microbes, such as bacterial species resident in the gut or lung as well as dormant viral species, i.e., cytomegalovirus (CMV) or Epstein-Barr virus (EBV), in combination with a balanced immune regulation, is central to achieve immune-protection. Emerging evidence suggests that immune responses primed to guard against commensal microbes may cause unexpected pathological outcomes, e.g., chronic inflammation and/or malignant transformation. Furthermore, translocation of immune cells from one anatomical compartment to another, i.e., the gut-lung axis via the lymphatics or blood has been identified as an important factor in perpetrating systemic inflammation, tissue destruction, as well as modulating host-protective immune responses. We present in this review immune response patterns to pathogenic as well as non-pathogenic microbes and how these immune-recognition profiles affect local immune responses or malignant transformation. We discuss personalized immunological therapies which, directly or indirectly, target host biological pathways modulated by antimicrobial immune responses.

## Background

Our understanding of the immune system stems, in great part, from studying the host response to infection, which in most individuals leads to the absence of clinical disease and establishment of highly apt immunological memory. The host immune response in relation to opportunistic pathogens as well as to the endogenous microbiota is pivotal to deter not only infectious diseases, yet also central to providing general physiological health (Manfredo Vieira et al., 2018; Mathieu et al., 2018a; Schachter et al., 2018). Chronic infections, particularly those which are primarily characterized by an asymptomatic intracellular life cycle, e.g., latent *Mycobacterium tuberculosis* infection (LTBI), hepatitis B virus (HBV) infection, *Chlamydia trachomatis* infection, cytomegalovirus (CMV) or Epstein-Barr virus (EBV) infections, present a unique premise to decipher the fine balance between protective host immune responses, immunopathology and full-fledged clinical disease. Nevertheless, while a chronic host immune response driven by pathogens may be protective against clinical disease, it may also elevate the risk of inflammation-induced dysplasia. The association of certain human leukocyte antigen (HLA) alleles which predispose individuals to a greater risk of harmful inflammation and disease (Mignot et al., 2001; De la Herran-Arita et al., 2013; Tafti et al., 2016; Matzaraki et al., 2017) play a central role in pro-inflammatory processes. We will first highlight some of the major neoplasia-associated infections of clinical relevance in the context of neoplasia and immune response modulation.

Although overt inflammatory responses play a major role in malignant transformation of host cells following an infection, it is a disbalanced immune responses, which contribute to drive malignant transformation. Thus, the local immunological milieu in tissue compartments forms the nature and magnitude of the host responses, i.e., frequencies of regulatory T cells (Tregs) vs. T-helper 17 (Th17) cells, amount of pro-inflammatory cytokines vs. anti-inflammatory cytokines, extent of neutrophilia and antigen-presenting-cell (APC) activation, among others. The second part of the review discusses potential host-directed interventional strategies based on existing translational and clinical knowledge of infection-induced inflammation, as well as cancer initiation/progression models.

## Pathogen-Driven Inflammation and Neoplasia: Existing Knowledge and New Insights

### Viral Pathogens and Immuno-Oncogenesis

Most infection-induced cancers worldwide are attributed to viral pathogens, possibly representing up to 80% of cases reported (Chang Y. et al., 2017). Although harbored by at least 90% of the world’s population, EBV causes malignant transformation only in a handful of individuals, which has been in part linked to the genetic variations in the infecting strain (Tzellos and Farrell, 2012). EBV-induced cancers, such as nasopharyngeal carcinoma (NPC) and B-cell lymphomas in the form of severe lymphoproliferative disease (LPD) following stem cell transplantation, non-Hodgkin’s lymphoma (NHL) as well as Hodgkin’s lymphoma (HL) are well documented (comprehensively reviewed in Saha and Robertson, 2011; Farrell, 2019). LPDs can also involve some populations of T cells (thus, manifesting as a T-cell lymphoma) and natural killer (NK) cells (Kim et al., 2017). The fact that patients with some cancer histologies/molecular profiles respond to immune checkpoint inhibitors (ICI), such as anti-PD-1, anti-CTLA-4, and anti-PD-L1 allows the study their impact on non-target T-cell populations (those not directed specifically against cancer-associated mutations or neoantigens), i.e., on CMV or EBV-reactive T cells. A clinical study with anti-PD-1 blockade in patients with lung cancer showed that EBV-specific T cells were not expanded during lung cancer treatment (Kamphorst et al., 2017). There is also a clinical trial currently underway to treat patients with EBV-positive NHL or other LPDs with EBV-specific cytotoxic T cells activated using antigen-pulsed dendritic cells in combination with nivolumab (anti-PD-1 antibody) (ClinicalTrials.gov identifier: NCT02973113). EBV-specific tumour infiltrating lymphocytes (TILs)/T cells have also been shown to mediate tumor killing *in vitro* as well as disease remission in patients with NPC (He et al., 2012; Li et al., 2015). HLA-B35, along with HLA-B2, -A2 and -A11 have been shown to be associated with a higher risk of developing post-transplant lymphoproliferative disease (PTLD) post solid-organ transplantation (Pourfarziani et al., 2007), while another study in Denmark showed that HLA-B45 and HLA-DR13 pose an increased PTLD risk (Vase et al., 2015). Indeed, a HLAB35-restricted epitope from EBV BZLF1 protein was previously shown to elicit strong cytotoxic T-cell responses (Tynan et al., 2005), while circulating IFN-γ+ CD8+ T cells in patients with PTLD were dominantly reactive to a HLA-B35-restricted epitope from EBV Epstein-Barr nuclear antigen 1 (EBNA1) (Jones et al., 2010). Interestingly, EBNA1 is also involved in downregulation of the HLA class I molecule to avoid immune surveillance (Levitskaya et al., 1995), while, more recently, the late lytic cycle associated EBV protein BDLF3 (recombinant EBV probable membrane antigen GP85) was shown to downregulate HLA class I and class II, CD54 (ICAM-1, important for cell trafficking and adhesion) and CD71 (transferrin receptor, necessary for iron homeostasis) (Quinn et al., 2015). EBV-derived IL-10 has been shown to induce pro-inflammatory polarization in human monocytes by STAT3 (signal transducer and activator of transcription 3) downregulation (Jog et al., 2018) and is more efficient than human IL-10 at inducing B-cell proliferation along with a high density of IL-10R1 expression on the cell surface (Yoon et al., 2012). The Cancer Genome Atlas Research Network published a comprehensive molecular analysis of gastric cancer (GC) samples to identify possible mutational signatures and found that an EBV-associated gene expression profile, particularly in tumor tissue isolated from the gastric fundus (upper part of the stomach) in line with a DNA hypermethylation pattern, formed a distinct clinical subtype of GC (Cancer Genome Atlas Research, 2014).

*Merkel cell polyomavirus (MCPyV)* is associated with most cases of Merkel cell carcinoma (MCC), considered an aggressive, though relatively rare, neuroendocrine skin cancer (DeCaprio, 2017; Miller et al., 2018). Interestingly, both MCC “virus-positive” or “virus-negative” are very immunogenic and elicit not only a CD8+ and CD4+ T-cell response (Iyer et al., 2011), but also a B-cell response against MCPyV T-antigen proteins (Paulson et al., 2010, 2017). A striking feature concerning the MCPyV is the fact that it can cause a lifelong, but relatively innocuous infection, even in people at an early age (DeCaprio, 2017).

*Merkel cell polyomavirus* has two transcriptional units coding four splices mRNAs encoding four proteins, which includes a large T-antigen (LT), 57kT, small T-antigen (ST) and ALTO, and also two viral coat proteins (VP1 and VP2) (Carter et al., 2013; Theiss et al., 2015). It is described that approximately 80% of all MCC contains clonally integrated copies of these virus, leading in mutations resulting in LT truncation with deletion of its DNA binding and helicase domains and 57 kT growth suppressing domain at the carboxyl-terminus loss (Shuda et al., 2008; Li et al., 2013). Typically, virus-positive MCC tumors express ST and truncated LT, supporting their role in the initiation and maintenance of MCC. Besides the presence of viral DNA and protein expression, tumor genomes are significantly different between virus-positive and virus-negative MCC. Indeed, “virus-positive” MCC contains few somatic mutations and copy number alterations. On the other hand, “virus-negative” MCC have a high frequency of DNA mutations, since these MCC are usually associated to UV damage (Wong et al., 2015; DeCaprio, 2017; Starrett et al., 2017). Another difference is that most “virus-positive” MCC still contains a non-mutated retinoblastoma suppressor gene (RB1), contrarily to “virus-negative” MCC, in which RB1 is usually mutated (Harms et al., 2016). The protein coded by this gene can restrict cell-cycle progression by inhibiting the entrance to S phase due to E2F family repression. Therefore, MCPyV MCC will fail to stop in G1/S and increase the proliferation rate of such cells (Borchert et al., 2014; DeCaprio, 2017).

There is a close relationship between MCC and other malignancies associated to immune cells, such as chronic lymphatic leukemia, multiple myeloma or non-Hodgkin lymphoma. Indeed there is an increased incidence of MCC in patients that also have one of the diseases mentioned above (Tadmor et al., 2011), probably due to the compromised immune responses in lymphoid malignancies. Indeed, these patients will probably have a higher propensity for colonization with MCPyV, which would then increase the incidence of MCC. Besides, patients with lymphoid malignancies are also frequently subjected to T-cell-suppressive therapies, that would influence MCPyV infection and the course of MCC (Tadmor et al., 2011; Brewer et al., 2012; Triozzi and Fernandez, 2013).

Various *human papilloma virus (HPV)* genotypes are generally associated with oncogenesis, although HPV-16 and -18 are implicated in the pathogenesis of several cancers in addition to cervical cancer (also HPV-31, -33, -35, -39, -45, -51, -52, -56, -58, -59), such as cancers of the oral cavity, oropharynx, and tonsils (Cogliano et al., 2011; Bansal et al., 2016). HPV-38, which is associated with skin cancer (Kocjan et al., 2009), interacts with the eukaryotic elongation factor 1 A (eEF1A) via the viral E7 protein to remodel actin fibers in the cytoskeleton and thereafter promoting aberrant cell proliferation, as shown in human keratinocytes (Yue et al., 2011). The double methylation of eEF1A Lys55 by the human methyltransferase-like 13 (METTL13) protein was recently found to be a crucial facilitator of mutant KRAS-driven oncogenesis in the pancreas and lungs (Liu et al., 2019). METTL13, which is also annotated as FEAT has anti-apoptotic functions (Liang et al., 2015) and has been linked to cirrhotic lesions in the human liver adjacent to hepatocellular carcinoma (HCC) tissue, suggesting a role in inflammation (Takahashi et al., 2011). The only approved vaccine against HPV-associated cervical cancer, Gardasil^®^, targets the L1 protein from HPV-6, -16, -11, and -18 and offers remarkable immunological protection of up to 5 years against HPV-6/-11-driven genital warts, as well as HPV-16/-18-linked cervical intraepithelial neoplasia grade 2 (CIN-2+) lesions (Harper et al., 2010). Patients with HPV+ head and neck cancer have been shown to have a higher number of PD-1+ CD8+ tumor infiltrative lymphocytes (TIL) infiltrating their tumors, compared to HPV-naïve patients further to experiencing better clinical outcomes following standard therapy (Kansy et al., 2017). In a clinical study testing TIL efficacy against HPV+ cervical cancer, nine patients with metastatic cervical cancer were given TIL, which in four of the patients had specific T cell recognizing HPV E6 and E7 peptides that were also detected post-infusion. Two patients who exhibited complete responses lasting between 15 and 22 months after T-cell therapy showed shared CD4+ T-cell reactivity to HPV E7_5__–__19_ (the first patient also has CD4+ T-cell responses to two other E7 peptides, including E7_9__–__19_) (Stevanovic et al., 2015), indicating the potential of HPV-specific T cells in mediating clinically meaningful responses in HPV malignancies. In another study, thirty patients with vulvar intraepithelial neoplasia infected with HPV-16 who were treated with long peptides from the HPV-16 E6 and E7 proteins (two patients received three vaccinations, while the remaining 28 patients were given four vaccinations) showed very favorable clinical results (9/30 patients with complete response at 12 months post-vaccination, which last a further 12 months – thus a total of 24 months) (Kenter et al., 2009). A cervical cancer-derived CD8+ T cell specific for an HLA-A2-restricted HPV-16 E6 epitope has also been shown to be enriched in the tumor compared with peripheral blood (Draper et al., 2015). Furthermore, the corresponding TCR was transduced into naïve T cells and tested for anti-tumor activity against several HPV+ cancer cell lines, resulting in target-cell elimination. These clinically relevant results are indicative of the potential anti-tumor functionality of HPV-specific T cells either directly or by means of modulating the local immunological milieu by molecular mimicry to potentiate disease control.

A link between immune evasion in cervical cancer cells, perpetrated by HPV, has been shown in the context of HLA class II molecules. The effect of IFN-γ on HPC+ cervical cancer cells has been shown to mobilize class II-associated invariant chain peptide (CLIP) and HLA-DMA into the cytoplasm while HLA-DMA dynamics (HLA-DM being a “molecular editor” for loading peptides into the MHC class II binding cleft) was not affected, although HLA-DR expression at the cell surface was increased (Zehbe et al., 2005). In line with this, IFN-γ treatment of HPV+ ME180 cervical cancer cells promoted immune recognition by a HLA-DR4-restricted CD4+ T-cell clone CCA1 (which is specific for a peptide derived from the HPV E7 protein) (Zehbe et al., 2005). Nevertheless, CLIP expression was not affected by IFN-γ treatment, hinting at alternate strategies by which HLA class II immune surveillance may be increased in HPV-associated malignancies.

The involvement *of hepatitis B and C viruses (HBV/HCV)* in the pathogenesis of HCC is also well-established. A combination of natural selection of antagonistic HLA class I viral epitopes and upregulation of PD-1 on infected hepatocytes contributes to immune evasion of HBC/HCV in the liver (Bertoletti et al., 1994; Cox et al., 2005; Golden-Mason et al., 2007). Virus-mediated counter-regulation of cholesterol metabolism in host cells by manipulating the subtilisin Kexin Isozyme-1 (SKI-1)/Site-1 Protease (SKI-1/S1P) pathway has been implicated in the establishment of a successful infection (Olmstead et al., 2012). This was further strengthened by the finding that pharmacological inhibition of the SKI-1/S1P pathway limited viral particle generation by reducing the amount of intracellular lipid droplet formation. A similar scenario has been observed in the infection of liver cells with dengue virus, another highly clinically relevant flavivirus, and it’s blockade with PF-429242, specific inhibitor of the SKI-1/S1P pathway (Hyrina et al., 2017). The HBV-encoded HBx protein has been previously shown to interact with the afore-mentioned eEF1A, which is a downstream target of the METTL13/FEAT enzyme and participates in remodeling of actin filaments in hepatoma cells by blocking eEF1A dimerization (Lin et al., 2012). Anti-PD-1 therapy with nivolumab induced strong anti-viral activity to the effect of reducing viral load by approximately 4-log, with the viral load reduced to undetectable levels in two patients (Gardiner et al., 2013). Similarly, anti-CTLA-4 blockade (tremelimumab) in patients with HCC and chronic HCV infection extended the time to disease progression, with almost 60 patients experiencing stable disease and in addition to dramatic reduction in HCV load (Sangro et al., 2013). IFN-γ-producing T cells, in response to HCV antigen exposure, were also detected in peripheral blood of some patients up to 300 days post tremelimumab treatment initiation (after at least three cycles had been completed). Apoptosis-inducing cytotoxic T cells with engineered with anti-HCV TCRs (directed against the non-structural proteins NS3 and NS5) as well as those targeting HBV are being developed (Balasiddaiah et al., 2017; Bertoletti et al., 2017; Kah et al., 2017).

*Kaposi’s sarcoma herpes virus (KSHV)*, presently known as human herpesvirus-8 (HPV-8), is the etiological agent of Kaposi’s sarcoma, a form of endothelial cancer which can manifest in mucosal tissue surfaces, i.e., lymph nodes and skin as well as two other lymphoproliferative disorders, namely multicentric Castleman’s disease (MCD) and primary effusion lymphoma (PEL) (Robey et al., 2011). Kaposi’s sarcoma (KS) was, in the 1980s and early 1990s, identified as a major co-morbidity of HIV/AIDS-related immunosuppression (Chang et al., 1994; Strickler et al., 1999). KSHV can dampen innate immune responses via several strategies (reviewed in Lee et al., 2010), simultaneously producing viral interleukin (IL) 6 which promotes the pathogenesis of MCD and PEL (Sakakibara and Tosato, 2011). Importantly, latent KSHV has been reported to upregulate host IL-6 production (by infected cells) triggered by the KSHV latency-associated nuclear antigen (LANA), hinting at an ongoing inflammatory response despite absence of clinical disease as well as in MCD and PEL (An et al., 2002). Central memory T-cell responses (IFN-γ) to the virus has been observed to the crucial even in the presence of sirolimus therapy (rapamycin, immunosuppressant) following renal transplantation (Barozzi et al., 2008). In a recent clinical study investigating the therapeutic efficacy of anti-PD-1 (nivolumab or pembrolizumab, anti-PD-1 antibodies) blockade in HIV-associated KS, six out of nine patients who received the treatment showed objective responses (five patients = partial responses; one patient = complete remission) (Galanina et al., 2018). Interestingly, some of the patients who responded to anti-PD-1 therapy (*n* = 4) displayed low PD-L1 levels in their tumors, coupled with low CD4 counts and high HIV burden.

Although not related to cancer, influenza vaccine-mediated narcolepsy development in susceptible individuals presents a very clinically relevant human modality pertinent to antigen cross-reactivity influenced by the restricting HLA elements in an individual (Mignot et al., 2001; De la Herran-Arita et al., 2013; Tafti et al., 2016; Bomfim et al., 2017). HLA-DQB1^∗^0602 confers protective immune responses against developing type 1 *diabetes mellitus* (T1DM), while the same individuals suffer a greater risk of contracting narcolepsy (Mignot et al., 1997, 2001; Siebold et al., 2004). Similarly, some HLA class I alleles, i.e., HLA-A11:01, HLA-C04:01, and HLA-B35:01, are implicated in the susceptibility of certain groups of individuals to develop narcolepsy (Tafti et al., 2016). HLA-DQB1^∗^0602 in association with HLA-DRB1^∗^1501/02/03 present a propensity to induce development of Th17 and Th1 cells, which is necessary for the control of bacterial infections at very early stages, but not later due to the effect it has on culminating in autoimmune pathology (more reflective of Th17 cells) (Mangalam et al., 2013). Asian populations have a higher frequency of the HLA-DQB1^∗^0602 allele, while West African have at least twice as more polymorphisms in the HLA class II allele pools compared to White Caucasians, which may have evolved to provide superior protection against the former populations against infections agents such as *Mycobacterium tuberculosis*, *Klebsiella* sp., *Leishmania* sp. (Mangalam et al., 2013). In contrast, HLA-DQB^∗^0601 is associated with resistance to developing autoimmune disease, based on studies in mice with experimental autoimmune encephalomyelitis (Mangalam et al., 2008), the murine model of multiple sclerosis (MS).

### Bacterial Pathogens, Inflammation, and Oncogenesis

*Helicobacter pylori* is perhaps the best described and most known oncogenic bacterial pathogen in relation to gastric cancer (GC) thus classified as a group I carcinogen (Wroblewski et al., 2010). Proteins produced by *H. pylori*, comprising the vac and cag families, induce molecular changes in stomach epithelial cells leading to malignant transformation (Wroblewski et al., 2010). Bacterial urease, on the other hand, promotes apoptosis of epithelial cells by direct binding to the HLA class II molecules (Fan et al., 2000). Downregulation of HLA class II molecules by *H. pylori* strain N6 via enhanced expression of CD300E and miRNA-4270 suppression in human macrophages was recently found to be an immune evasion strategy employed by this pathogen (Pagliari et al., 2017). *H. pylori* VacA (from strain CCUG17874) was previously shown to interfere with the synthesis and presentation of nascent HLA class II molecules (Molinari et al., 1998), as well as the interaction between B7-H2 and CD28 on CD4+ T cell to trigger immune activation marked by early IL-17 production to control infection (Lina et al., 2014). Thus, deterring CD4+ T-cell responses appears to play an important role in *H. pylori*-mediated immune evasion, but the same effect may play potentiate anti-cancer responses. An interesting example being the observation of IFN-γ+ CD4+ T cells responding to HLA-DRB1^∗^1501-restricted *H. pylori* hemagglutinin (HpaA_88__–__100_) in *H. pylori*-infected patients with GC who exhibited less severe disease (Chen et al., 2013).

The local immune responses in the stomach to *H. pylori* has also been shown to be strain-dependent, augmenting the release of a panel of pro-inflammatory cytokines, such as IL-6, TNF-α, IL-12, and IL-1β (Andres et al., 2011). Also, the suppression of microRNA Let-7b expression in gastric cells by *H. pylori* infection promotes TLR4 expression and downstream Myd88 activation to trigger NF-κB-mediated immune responses (Teng et al., 2013). The finding that *H. pylori* infection leads to upregulation of vitamin D receptor (VDR) expression in gastric epithelial cells and extracellular supply of vitamin D3 is able to improve intracellular bacterial killing (Guo et al., 2014) indicates that a subpopulation of patients may be able to control bacterial infection via vitamin D-associated mechanisms. A recent review of clinical trial data suggests that vitamin D may have a protective effect in cancer although further controlled real-world evidence is required to strengthen this stance (Grant, 2018).

*Escherichia coli*-derived colibactin and the risk of colon/colorectal cancer (CRC) has been proposed due to the DNA alkylating nature of the bacterial toxin. The strain of *E. coli* which produces colibactin is prevalent in the human gut, but not everyone with colibactin + *E. coli* develops malignancies. An earlier preclinical study showed that the colibactin gene is encoded in the *pks* genomic island of certain *E. coli* strains, which have also been recovered from some patients with CRC (Dalmasso et al., 2014). Furthermore, using cell culture and a mouse model of human CRC, pks + *E. coli* negatively affects p53 SUMOylation and, thereafter, the structural integrity of the p53 protein leading to DNA breaks. Infection of cells (as well as mice) with pks + *E. coli* also induced the production of growth factors, i.e., HGF, FGF, and GM-CSF associated with tumor outgrowth and poor outcome. Enteropathogenic *E. coli* (EPEC) which produces Shiga toxin, the causative agent of haemolytic uraemic syndrome (HUS), may also be involved in the pathogenesis of CRC based on the findings of a clinical study published in 2015 (Magdy et al., 2015). *Citrobacter rodentium* infection of the mouse colon, which represents similar pathological features to EPEC infection and intestinal inflammation in humans, has been shown to promote Th17 induction with the involvement of the resistin-like molecule alpha (RELMα) (Osborne et al., 2013).

In their meta-analysis of 22 clinical studies, Zhu and colleagues further strengthened the existing link between *C. trachomatis* infection and the occurrence of cervical cancer (Paavonen, 2001), while identifying co-infection with HPV to promote a higher risk of developing malignant disease. A 2019 clinical study showed that women with serum antibodies against the Pgp3 protein of *C. trachomatis* suffer a twofold risk of developing ovarian cancer (Trabert et al., 2019). Pgp3 is a plasmid-encoded virulence factor which can form a complex with human cathelicidin (also known as LL-37) to induce IL-6 and IL-8 production in neutrophils (Hou et al., 2019). Mechanistic studies using cell culture systems have provided empirical proof that infection with *C. trachomatis* induces DNA double-strand breaks (DSBs) in cells while impairing the repair process by inhibiting recruitment of ataxia telangiectasia mutated kinases (ATM) and p53-binding protein 1 (53BP1), further to inducing production of the epidermal growth factor (EGF), which is implicated in dysplasia (Chumduri et al., 2013; Patel et al., 2014).

To date, no direct link has been established between *Mycobacterium tuberculosis* (*Mtb*) infection and oncogenesis in the lungs. However, some existing clinical and translational evidence suggest that *Mtb* infection-induced fibrotic lesions in the lungs may contribute to malignant transformation, partly due to genetic aberrations caused by the local inflammatory response comprising an armament of IFN-γ, TNF-α, IL-12, IL-18, IL-17, and IL-6 to clear the pathogen (Kaufmann et al., 2014; Zumla et al., 2015; Nikitina et al., 2018). Mutations in the epidermal growth factor receptor (EGFR) gene, associated with lung adenocarcinoma pathogenesis, have been linked to the presence of old pulmonary tuberculosis (TB) lesions in patients with lung cancer (Luo et al., 2012). In agreement with this, the presence of old pulmonary TB lesions has been shown to be an independent predictor of shortened survival among patients with squamous cell carcinoma of the lung (SCC) (Zhou et al., 2013). A 2006 report showed that 25% of patients in a New York City study cohort with an underlying cancer diagnosis, i.e., lung cancer, Hodgkin’s and NHL, head and neck cancer or leukemia, died within three months of being diagnosed with pulmonary TB (Kamboj and Sepkowitz, 2006). Taiwanese patients with various solid cancers (particularly lung cancer) and TB, compared to those who did not have cancer, exhibited a significantly lower anti-TB treatment success rate (43 vs. 75.8%) and almost four times the mortality rate (48.4 vs. 13.9%) (Chiang et al., 2009). *Mtb* infection has been shown to induce DSBs in host macrophages, reminiscent of pre-apoptotic DNA fragmentation (Castro-Garza et al., 2018), hinting at an added mechanism of promoting malignant transformation of host cells.

Some bacterial pathogens, such as *Salmonella*, *Klebsiella*, and *Yersinia* sp., which are etiological agents of enteric infection, have been associated with downregulation of the HLA-B27 allele in PBMCs of infected patients (Kirveskari et al., 1999). These pathogens have also been associated with the pathogenesis of ankylosing spondylitis (AS), particularly in HLA-B27+ individuals (Martínez et al., 2004). HLA-B27 expression is a risk factor for AS and patients with AS have an increased risk for developing hematological malignancies, colon, bone as well as prostate cancer (Chang C.C. et al., 2017). An association between HLA-B27 and immune responses against ovarian cancer – associated antigens has been reported (Szender et al., 2016). Taking these into consideration, it would be clinically relevant to investigate the relationship between bacterial infection at mucosal surfaces, local immune responses therein and the modulation of HLA alleles in relation to neoplasia. Although beyond the scope of this paper, a recent genome-wide association study (GWAS) in more than 200,000 individuals of European ancestry provided evidence of susceptibility loci in the HLA class I and class II alleles (Tian et al., 2017) to various infections (bacterial, viral, and fungal), thus paving the way for future high-throughput large-scale studies to better understand the fine details of infection and immunomodulation.

### Helminths in the Inflammation-Induced Oncogenesis

Besides the role of viral and bacterial pathogens as well as the microbiome discussed above, several helminth infections – mainly affecting the developing world – are an important cause of infection-induced inflammation and oncogenesis. *Schistosoma haematobium*, commonly known as the urinary blood fluke, is a parasitic flatworm prevalent in Sub-Saharan Africa and the Middle East and recognized by the IACR as a human carcinogen (Cogliano et al., 2011). Eggs deposited in the bladder wall has been shown to induce chronic inflammation following long-term interaction with the host’s immune cells (Ishida and Hsieh, 2018). Furthermore, *S. haematobium*-induced inflammation can result in KRAS mutations leading to oncogenic transformation of bladder cells (Botelho et al., 2013), while parasite-derived antigen preparations can directly induce inflammation leading to dysplasia in otherwise normal and healthy mice (Botelho et al., 2011). Similarly, the Southeast Asian liver fluke *Opisthorchis viverrini* and Chinese blood fluke *Clonorchis sinensis* have also been shown to perpetrate liver and bile duct cancer by means of establishing chronic inflammation (Sripa et al., 2007, 2012; Kim et al., 2016). *O. viverrini* also secretes an important mitogenic factor, namely granulin-like *Ov-*GRN-1, which promotes aberrant growth of host cells in the bile duct further to activating Myd88-dependent NF-κB-driven inflammation (Sripa et al., 2012). During the chronic phase of *C. sinensis* infection, a disrupted balance between pro- and anti-inflammatory immune responses (Th1 suppression, Th2 increase) leads to DNA damage and neoplastic transformation of host cells (Kim et al., 2016). As such, the immunological axes involved in helminth-induced oncogenesis are akin to those observed in cancers with bacterial and viral pathogens.

## The Microbiome in Inflammation-Induced Neoplasia

The role of the microbiome, especially in the gut, play a quintessential role in maintaining physiological balance and homeostasis in health and disease (Roy and Trinchieri, 2017). Metabolites derived from gut bacteria, such as butyrate, are essential for maintaining gut barrier function (Zheng et al., 2017), which is necessary for deterring unwanted inflammation. The gut microbiota is also directly involved in drug metabolism and control of drug-associated toxicity (Zimmermann et al., 2019). Dysbiosis of the lung microbiota is implicated in the development of autoimmune pathology (O’Dwyer et al., 2016), while immune activation of gut fungal microbiota-specific T cells can influence and alter immune responses in the lung (Bacher et al., 2019). Among the constituents of the microbiome which often emerge in clinical studies are *Bacteroides* and *Prevotella* species which, in addition to the gut, are also members of the lung microbiome (Mathieu et al., 2018b; Pragman et al., 2018). The former has been associated with desirable clinical outcomes in patients with metastatic melanoma who undergo anti-CTLA-4 therapy (Chaput et al., 2017). Translational and clinical studies have linked an abundance of *Prevotella* sp. in the gut to improved protection to influenza infection (Lee et al., 2019), clinical TB (Maji et al., 2018), and general immunological homeostasis in the airways (Huffnagle et al., 2017), thus providing a link between this commensal and pulmonary immune equilibrium. *Prevotella* abundance has been reported in patients with ulcerative colitis (UC) who are also smokers (Pascal et al., 2017), in agreement with a previous study which also found an abundance of *Bacteroides* (Lucke et al., 2006). In contrast, pediatric patients with new-onset Crohn disease (CD) (thus, treatment-naive) do not have a link to *Prevotella* in the gut microbiota but, however, display a reduced abundance of *Bacteroidales* (Gevers et al., 2017). It is also relevant to refer to another recent finding of a megaphage that specifically targets *Prevotella* species in the human gut (Devoto et al., 2019), warranting further investigation in relation to induction of intestinal inflammation and neoplasia.

Mechanistic studies performed in preclinical models have shed light on some hitherto unknown immune-related mechanisms driving – potentially also contributing to neoplastic transformation of tissue – with a link to the local microbiota. IL-17B production by APCs following exposure to bacterial outer membrane vesicles (OMVs) has recently been shown to enhance the development pathogenesis of bleomycin-induced pulmonary fibrosis (PF) (Yang et al., 2019). Most strikingly, the OMV which appeared to be most prominently promoting fibrotic transformation in the alveoli of mice derived from *Bacteroides* and *Prevotella* species. The lung microbiome in mice – but not including *Bacteroides* and *Prevotella* – were shown to augment IL-1β and IL-23-driven (also involved in IL-17 production) T-cell pathology which promoted mutant KRAS and p53 lung adenocarcinoma development (Jin et al., 2019). This pathway was eventually mapped to the activation of Vγ6Vδ1+ T cells and their local production of IL-17 perpetrating malignant transformation. In another study that investigated the role of TCR γδ T cells in celiac disease, a specific population of intraepithelial lymphocytes (IEL) comprising cytolytic Vγ4Vδ1+ T cells recognizing butyrophilin (BTLN) 8/3 were shown to be decreased following gluten-induced inflammation concomitant with BTLN8 downregulation (Mayassi et al., 2019). Instead, a new subset of Vδ1+ T cells, which did not recognize BTLN8/3, occupied the same IEL niche. Provision of a gluten-free diet allowed for recuperation of BTLN8 expression, although the host-protective Vγ4Vδ1+ IEL populations could not be rejuvenated. Celiac disease has been linked to predisposing patients to a higher risk of developing esophageal cancer (Han et al., 2015), thus making it a relevant model to study early events in inflammation-induced neoplasia.

Commensal bacteria which trigger IL-17 and IL-22 production in the gut may in fact do so via Mincle-Syk kinase and C-type lectin 4e (Clec4e) signaling in dendritic cells (DCs), as recently shown in murine Peyer’s patches (Martínez-López et al., 2019). The signaling pathway, in response to sensing of microbiome-associated molecular patterns, produce IL-6 and IL-23p19, which in turn promote the development of Th17 and the production of IL-22 from type 3 innate lymphoid cells (ILC3) in the intestines. Aberrations in the Mincle-Syk pathway led to reduced gut barrier integrity, bacterial translocation to the liver and promotion of inflammation, in addition to compromised production of IgA by resident plasma cells. Adhesion-mediated endocytic uptake of antigenic cargo derived from the cell wall components of commensal gut bacteria by intestinal epithelial cells (IECs) appears to induce Th17 development in mice, requiring the activity of cell division control protein 42 homolog (CDC42) (Ladinsky et al., 2019). Thus, this system postulates one important mechanism by which microbiota-directed T-cell responses are primed in the gut and how this may play an induction of deleterious inflammation in disease.

Elevated levels of CD4+ T cells which produce IL-17 (Th17 subset) in peripheral blood of patients with type 2 *diabetes mellitus* (T2DM) has been recognized as a major contributor to chronic inflammation in these individuals (Jagannathan-Bogdan et al., 2011). Importantly, perturbation in the gut microbiome is very intimately linked to DM development (Harsch and Konturek, 2018), thus making the IL-17 axis a very relevant accomplice in this regard. More recently, a meta-analysis of 121 cohorts comprising 20 million individuals from across the globe confirmed that DM predisposes patients to developing various cancers, generally putting women more at risk than men (Ohkuma et al., 2018). A clinical study reported in 2016 showed that patients with T1DM and chronic periodontitis (oral bacterial infection) carry polymorphisms in the *IL17A* gene perpetrating exaggerated cytokine production and pathology (Borilova Linhartova et al., 2016). Patients with T2DM and pulmonary TB also exhibit high levels of serum IL-17A, in addition to TNF-α and IFN-γ, concomitant with disease severity in the lungs and reduced functionality of CD8+ cytotoxic T cells and NK cells (Kumar et al., 2013, 2015). These findings hint at a role for IL-17A in predisposing individuals with DM, particularly those harboring infections which trigger IL-17 responses, to neoplastic transformation.

Another interesting point was raised by Yoshimoto et al. (2013), that an obesity-associated distinct microbiome appears to be associated with higher incidence of liver cancer; the increase in deoxycholic acid (DCA) – associated to microbiome alterations, as a result of obesity – may provoke a senescence-associated secretory phenotype (SASP) in hepatic stellate cells, and subsequently increase the production of inflammatory molecules and tumor-promoting factors in the liver associated with an increase in HCC development after exposure to chemical carcinogens (Yoshimoto et al., 2013). Another study showed evidence of SASP signs in hepatic stellate cells in non-alcoholic steatohepatitis (Sun and Karin, 2012), demonstrating that a broad array of different factors may affect the microbiome and its complex impact on harmful or protective immune responses. Preferential colonization of mucosal tissues by certain microbiome-associated bacterial species – influenced by infections, drug intake, dietary changes and other lifestyle practices, e.g., smoking, breastfeeding (Victora et al., 2016) – can trigger unexpected immunological programs which can either promote neoplastic disease or offer protection. The microbiome does not only impact on the clinical outcome of patients with cancer treated with checkpoint inhibitors, yet also in long term survival of patients with pancreatic cancer. A high tumor microbiome diversity appears to be associated with immune activation; even a distinct intratumoral microbiome signature, defined by *Pseudoxanthomonas*, *Streptomyces*, *Saccaropolyspora*, and *Bacillus clausii*, has been shown to be associated with increased survival in patients with pancreatic cancer (Riquelme et al., 2019). In contrast to these beneficial microbiome signatures, bacterial or fungal species may drive harmful inflammation: *Saccaropolyspora rectivirgula* drives in general a pro-inflammatory environment, including hypersensitivity pneumonitis (Kim et al., 2010): the *mycobiome* (i.e., fungal commensals or pathogens) impacts on PDAC development mediated via (intratumoral) *Malassezia* that drives PDAC progression via complement activated inflammation (Aykut et al., 2019).

Among the currently discussed strategies to manipulate the microbiota, particularly in the gut and possibly also in the lung, is the use of CRISPR technology as well as the use of bacteriophages to select for preferential colonization of tissue with bacteria, fungi, perhaps in conjunction with certain bacteriophages, to promote host-beneficial immuno-physiology (Lee et al., 2018). Another use of CRISPR has also been to study the horizontal gene transfer between bacterial species comprising the microbiota and potentially pathogenic genetic islands that can perpetrate strong inflammatory responses and tissue transformation (Munck et al., 2018).

## Molecular Mimicry of Pathogen- and Host-Derived Targets: Implications in Anti-Cancer Immune Responses

Early work using T-cell lines specific for the melanoma-associated antigen Melan-A/MART-1_27__–__35_ HLA-A2 epitope (AAGIGILTV) showed cross-reactivity with an *E. coli* methionine synthase-derived peptide (AAGIGIIQI) and a peptide from the HSV-1 glycoprotein C (GIGIGVLAA), eliciting lysis and IL-2 production (Loftus et al., 1996; Carrabba et al., 2003). The binding of *Mtb*-derived epitopes with mimicry to host peptides to common HLA class I and class II molecules has also been shown using bioinformatic analysis (Chodisetti et al., 2012). This concept has now been reawakened in line with clinical biomarkers and immunotherapy in cancer, with a seminal article dissecting the immunological characteristics of long-term survivors of pancreatic cancer in whom polyclonal, neoantigen-specific CD8+ T-cell responses along with a high load of neoantigens emerged as the quintessential determinant of protection against disease progression (Balachandran et al., 2017). The authors also incorporated data linking the high quality of neoantigen-directed TCRs – which had not been negatively selected during disease pathogenesis due to their cross reactivity to microbial peptides (Balachandran et al., 2017). These cross-reactive T cells were found in blood and tumor tissue. This appears to be biologically (and immunologically) plausible, as it is in the host’s interest to fight pathogens and, therefore, not to remove these TCRs from the memory pool.

Another study, based on mathematical modeling of neoantigen characteristics and mimicry of pathogen-derived targets, showed that increased dissimilarity of neoantigens to (non mutant) target sequences increased the possibility to produce meaningful immuno-protective responses after immune checkpoint blockade therapy – designated as “neoantigen fitness” – further to binding to TCRs which also recognize infectious disease-related targets (Luksza et al., 2017). Thus, the concept of molecular mimicry which allows for pathogen- and host-reactive T cells to elicit immune responses may be crucial in mediating clinically relevant responses in cancer as well as infectious diseases.

## Infection, Inflammation, and Neoplasia: Implications for Personalized Immunotherapy

Targeting the immune system at early stages of infection may be detrimental, as the developing anti-pathogen response mounted by the host is crucial for augmenting innate and adaptive immune responses to control the pathogen’s replication as well as to form long-term immunological memory. The window of opportunity for modulating the host immune responses lies in clinically intervening when the inflammatory milieu becomes overt and perpetrates unnecessary tissue pathology – potentially leading to oncogenesis. Particularly, this time point is almost impossible to capture in humans, with some exceptions, as tissue transformation in patients with *H. pylori* (Dore et al., 2018) or EBV infection (post-transplantation) (Shannon-Lowe et al., 2017), which can be monitored more closely using appropriate diagnostics and immunological testing, respectively. Primarily, the timing of intervention herein is of utmost importance. For instance, in patients with drug-sensitive pulmonary TB who undergo the standard 6-month antibiotic therapy, IL-6 overproduction at diagnosis (serum levels) can be predictive of individuals who will sustain severe lung damage at the end of treatment – albeit successfully clearing the pathogen from the lungs (Nagu, 2017). In contrast, patients who produce too little IL-6 are more likely to succumb to TB and die within the first 2 months post-diagnosis and after initiating treatment. Thus, and as previously shown, IL-6 is necessary to control *Mtb* infection in patients, but overt circulating amounts of the cytokine may be deleterious and can, therefore, be considered for therapeutic targeting. Therefore, aberrant inflammatory processes which do not result in optimal clinical outcomes offer the ideal Achilles heel for intervention. Along these lines, platforms such as spatial transcriptomics (ST) (Berglund et al., 2018) and immunohistology-based mathematical modeling (hyperspectral cell sociology) (Enfield et al., 2019) can be useful in determining areas in diseased tissue where inflammation occurs and how this affects neoplastic transformation at the gene expression level even before pathological features and clinical symptoms can be observed.

Another important fact is the existence of microbiome variations due to geographical and ethnical differences, which should also be considered in personalized therapy. Indeed, there are several studies reporting significant variations in microbiome composition in healthy individuals from different ethnicities (Nasidze et al., 2009; Nam et al., 2011; Yap et al., 2011; Yatsunenko et al., 2012; Gupta et al., 2017). These differences may also predispose individuals for some malignancies, such as inflammatory bowel disease (IBD), or increase the risk of a viral infection with oncogenic potential. An example is association between EBV and NPC, which is endemic in southern Chinese population, with special incidence in individuals of Cantonese origin. Most likely, genetic susceptibility and environmental factors, such as the consumption of dietary nitrosamines, play a role in EBV and NPC incidence (Tsao et al., 2017).

### Using Immunological Effectors as Host-Directed Therapeutics

Recent evaluation of human T-cell responses to opportunistic pathogens has also revealed some interesting insights into immune priming and the risk of tissue pathology. Bacher et al. (2019) showed that CD4+ T cells which produce in response to *Candida albicans*, a commensal fungus which can cause opportunistic infections, readily cross-react with at least 30 other fungal species, based on assessment of peripheral blood-derived T-cell responses. Importantly, IL-17+ CD4+ T cells which recognize *C. albicans* antigens and cross-react with *Aspergillus fumigatus*, were identified to be specifically expanded in tissue samples from patients with airway disease (chronic obstructive pulmonary disease, asthma, and cystic fibrosis). The same cell subset was found to be increased in blood from patients with Crohn’s disease, indicating their potential to travel to the lung and promote pulmonary inflammation. Effector memory CD4+ T cells which arise early in development and produce tumor necrosis factor (TNF-α) have also been shown to promote intestinal tissue growth, which is compromised in prematurely born infants (Schreurs et al., 2019).

For instance, influenza-specific tissue-resident memory (TRM) CD8+ T cells are indispensable for protection to full-fledged flu-associated pathology in the lung (Pizzolla et al., 2018). TRM cells are likely to be established in non-lymphoid tissue compartments and not even detected in blood, meaning that the highly specialized TCR repertoire in tissue differs from that seen in peripheral circulation. Indeed, TIL from human cancer tissue have an arsenal of TCRs which are specific for viral antigens, i.e., influenza CMV and EBV, but also react to cancer neoantigens (Simoni et al., 2018; Scheper et al., 2019). Thus, there is a high chance that a ‘pre-wired’ matrix of the tissue-resident TCR repertoire might participate in orchestrating pathology and disease due to molecular mimicry between immunogenic epitopes of microbes and host-associated antigens. One preclinical study showed that influenza-primed T-cell responses were able to recognize the overexpression of host proteins by the 3LL lung tumor cell line and confer protection against lung cancer development in a murine model (Iheagwara et al., 2014). TRM CD8+ T-cells have been shown to express a similar gene program as compared to TIL (Milner et al., 2017). These TRMs, characterized by a specific transcription factors (Hobbit, Blimp-1, Notch, Runx3) (Milner et al., 2017) are believed to reside in non-lymphoid tissues in order to be able to rapidly respond to infectious pathogens – or to cancer (Kumar et al., 2017) TRMs require mitochondria dependent lipid oxidation (Pan et al., 2017), a pathway that is not properly functioning in chronic inflammation, chronic infection as well as in cancer lesions, due to the immune-suppressive environment and a “frozen,” non-functional chromatin state (Philip et al., 2017), that appears to be at least in part be reversible (Li et al., 2019). The transcription factor Bhlhe40 maintains immune fitness in TRMs and in TIL, where a subpopulation of stem-cell like TCF1+ TIL have been shown to be responsive to checkpoint inhibition – and may indeed by responsible for clonal expansion in response to pathogens or cancer cells. Of note, checkpoint inhibitors are thought to revert dysfunctional *in situ* T-cell responses leading to clinically relevant anti-tumor responses (Pauken et al., 2016). However, clinically relevant immune responses were associated with recruitment of new T-cell clones accumulating into cancer lesions, since pre-existing (exhausted) *in situ* T-cell clones could not be reverted by checkpoint inhibitors, most likely due to “fixed” epigenetic imprints (Kurtulus et al., 2019; Yost et al., 2019). A “fixed” epigenetic landscape appears also to be associated with T-cell phenotypes (Ghoneim et al., 2017) that can be manipulated by affecting the transcriptional regulator Tcf7/Tcf1 (Kurtulus et al., 2019) or Bhlhe40 (Mehta et al., 2017) promoting and maintaining TRMs and tumor-infiltrating immune cells by increased mitochondrial metabolism (Li et al., 2019), a situation that can be also be achieved using the genetic modifier HDAC (histone deacetylase inhibitor tubastatin A) or fatty acid acetate by affecting (Bhlhe40-negative) T-cells leading to increased IFN-γ production (Li et al., 2019).

The pathogenesis of IBDs, such as CD and UC, have also been attributed to arise from dysregulated host responses to microbial products, i.e., increased prevalence of gut microbiota-derived sphingolipids such as ceramide and induction of pro-inflammatory responses locally (Bryan et al., 2016; Franzosa et al., 2019). Bacterial strains from the gut microbiota of a patient with UC have also been shown to induce Th17 cells in germ-free mice following oral inoculation (Atarashi et al., 2015). As IBDs pose a high risk for CRC development (Althumairi et al., 2016), therapeutic interventions manipulating the microbiome and inflammation may represent a viable option to prevent malignant transformation. Along these lines, α4β7 integrin-expressing regulatory T cells (Tregs) can be used a cellular product to neutralize deleterious local inflammation to improve disease outcomes (Goldberg et al., 2019). Since Tregs from patients with IBDs express low levels of α4β7, a combination of rapamycin (sirolimus) and all-trans retinoic acid (ATRA) can be used to generate α4β7-expressing Tregs *in vitro* for cellular therapy. Pertaining to resolution of intestinal inflammation and antimicrobial defense, administration of BT-11, a locally active immuno-modulatory drug which enhances oxidative phosphorylation in immune cells, was shown to potentiate Treg induction and suppression of Th1 as well as Th17 responses in the murine model of *C. rodentium*-driven intestinal inflammation without compromising anti-*C. rodentium* immunological memory and clearance (Leber et al., 2019). Importantly, BT-11 targets the lanthionine synthetase C-like 2 (LANCL2) pathway, which happens to be central to inducing IL-10 production and amelioration of overt inflammation during infection, as shown in the context of influenza virus (Leber et al., 2017) and *H. pylori* (Leber et al., 2016) infections. These findings highlight LANCL2 as a host-directed therapy (HDT) target, which is expressed in the cell membrane of immune cells. Whether this approach would concomitantly promote exacerbation of an underlying bacterial infection in humans has to be formally tested in a suitable preclinical setting prior to clinical evaluation in patients.

**FIGURE 1:**
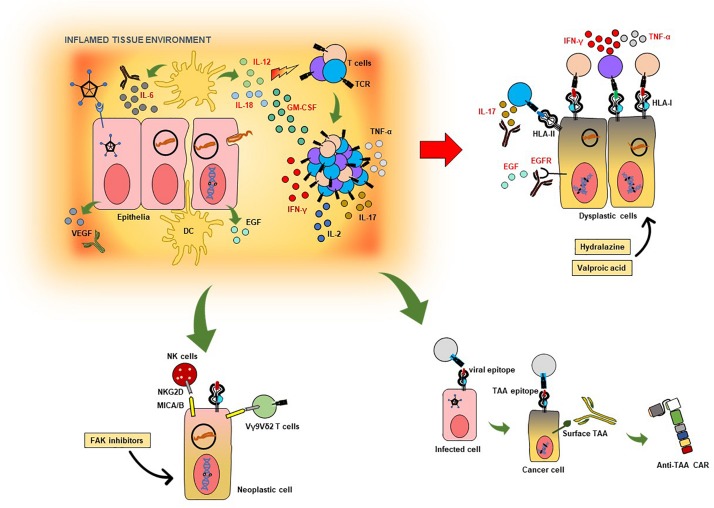
Targeted immunotherapeutic strategies against infection- and inflammation-induced neoplasia. The schematic shows some of the possible strategies which can be pursued in developing targeted host-directed immunotherapies based on biomarkers in infection-induced inflammation and oncogenesis. Cells infected with viruses or bacteria may induce physiological and anatomical changes in tissue, leading to reduced barrier functions and tissue integrity. This contributes to the overall local immunological milieu associated with the production of pro-inflammatory cytokines, as IL-6, IL-18, IL-12, and GM-CSF. In some cases of viral infection, VEGF production is also observed, which leads to neovascularization and poses a risk for malignant transformation. The same is true for EGF release by host cells in some bacterial infections. The ensuing T-cell activation culminates in production of IFN-γ, TNF-α, IL-2 as well as IL-17 in several infections – which can be pathogenic and contribute to chronic inflammation and neoplasia. To the right of the schematic are dysplastic cells, which can present antigenic epitopes to T cells, some of which may be protective (HLA class I-restricted pathogen- or host cell-derived structures), while others may exacerbate the IL-17 response (HLA class-II-restricted Th17 epitopes). Excess IL-17 as well as IL-6 can be neutralized using therapeutic antibodies which are clinically licensed. VEGF neutralization, as well as EGFR blockade, may be clinically useful in halting disease progression due to aberrant inflammation induced by infection. Hydralazine and valproic acid, both of which are clinically approved medications, can increase HLA class I expression in host cells, thereby augmenting and enhancing CD8+ T cell-mediated immune control. FAK inhibitors may be able to increase MICA/B expression in neoplastic cells (as well dysplastic ones) to improve immune surveillance by TCR Vγ9Vδ2 T cells and NK cells expressing the killer receptor NKG2D. Virus-induced T-cell responses against certain host-cell epitopes may educate T cells to subsequently recognize tumor cells; such T-cell populations may be used in active cell therapies and the nominal (antigen-specific) TCR may be utilized for transfer into recipient surrogate immune cells. Host cell surface molecules associated with malignant transformation induced by infection and inflammation, if recognized by circulating as well as tissue-associated antibodies, may be used as as templates for inducing ADCC as well as viable targets for biologically and clinically relevant chimeric antigen receptors (CARs) transgenically expressed by immune effector cells.

Killer receptor NKG2D-expressing immune effector cells such as Vγ9Vδ2 T cells, NK cells and some populations of TCR αβ CD8+ cytotoxic T cells which recognize the HLA class I-like molecules MICA/B, represent an essential component of the immune system’s arsenal against targeted elimination of diseased tissue, i.e., transformed cells in cancer and infectious diseases (Huergo-Zapico et al., 2014). For example, the FAK/Src pathway has been shown to trigger downregulation of the MICA in cells, which is reversible with the use of focal adhesion kinase (FAK) inhibitors (Moncayo et al., 2017). Although MICA/B were recently described to be localized intracellularly in many tumor cell types (Ghadially et al., 2017), inhibition of FAK may be able to increase its expression on the cell surface to promote cell-mediated cytotoxicity. At the transcriptional level, miR-10b has been shown to downregulate MICB expression in tumor cells, which is also reversible (Tsukerman et al., 2012). HBV, via upregulation of the transcription factors GATA2 and GATA3 – which is also involved in Th2 and ILC2 development in humans (Wan, 2014), can force the downregulation of MICA/B in hepatoma cells to escape NK-cell surveillance (Guan et al., 2016). As mentioned in the first part of this review, pharmacological inhibition of FAK, with the clinical candidates GSK2256098, defactinib and BI-853520, which are already in several clinical trials involving patients with solid and hematological malignancies [Clinical Trials.gov identifiers NCT00787033, NCT01778803, NCT03727880, NCT01951690, NCT01943292, NCT01138033 (Doi et al., 2019)], may also augment NKG2D-mediated immunomodulation. Figure 1 is a schematic representation of how these above-mentioned pathogen-directed immune responses may be used for HDT for timely intervention after host responses to infectious agent are detected.

### Pharmacological Agents for Therapeutic Immunomodulation in Infection and Neoplasia

Small molecules, such as valproic acid (VPA), a histone deacetylase inhibitor (HDI) and hydralazine may be used as adjuvants to improve immune responses in HPV+ patients and therefore may be a viable option due to the drugs’ capacity to upregulate HLA class expression in infected cells and CD8+ T-cell responses against them (Mora-Garcia Mde et al., 2006). Vorinostat, another HDI currently in clinical trials for cancer treatment (Bubna, 2015), along with VPA can actively inhibit *Mtb* growth in human cells and may shorten the treatment period with conventional first-line anti-TB drugs (Rao et al., 2018). Studies in mice suggest that carboplatin-derived cancer drugs exert their effects also via autophagy (Michaud et al., 2011), which facilitates anti-bacterial immune responses and clearance (Jo et al., 2013; Kaufmann et al., 2014). It may, therefore, be possible to investigate the immunological profile of cells isolated from platinum-treated patients with cancer for their responsiveness to pathogen-derived antigens and whether these cells can be used for therapeutic purposes. Rifampicin, which is a first-line anti-TB drug, has shown anti-angiogenic properties in preclinical studies, with inhibitory effects on human liver cancer cells and sarcoma cells (Shichiri and Tanaka, 2010), further to decreasing HCC to only a single occurrence in six patients who were HCV+ in over 97 months (8 years) (Shichiri et al., 2009). Since rifampicin is used not only to treat mycobacterial infections, but also those caused by meningococci, staphylococci and enterococci (Forrest and Tamura, 2010), the immunomodulatory properties of this drug may apply across the gastrointestinal and pulmonary organ systems. Based on experience in cancer treatment, low-dose cyclophosphamide-induced immunological fine-tuning by modulating CD8+ T and regulatory T-cell activity may also be useful in changing the immune milieu in immunocompetent individuals (Sistigu et al., 2011).

With regard to helminth infections, praziquantel has shown very good efficacy particularly in women and children based on the WHO-recommended parasite egg reduction rate (ERR) to measure control of parasite burden (Sayasone et al., 2017; Kabuyaya et al., 2018). While *S. haematobium* and *O. viverrini* infections exhibit better treatment outcomes, the treatment efficacy is somewhat reduced in the context of *C. sinensis*, with repeated doses required (Hong et al., 2001). This also increases the chances of drug resistance while not addressing the existing chronic inflammation in patients. Furthermore, praziquantel on its own can induce neutrophil activity in the bladder after treatment of patients with *S. haematobium* infection (Stecher et al., 2017). Allicin, a sulfur-containing compound extracted from garlic and available over-the-counter, was able to reduce inflammation as well as parasite burden albeit to a lesser extent than praziquantel in a mouse model of schistosomiasis (Metwally et al., 2018). Interestingly, allicin was also shown to reduce the viability of human CRC, breast and lung cancer cell lines in a dose-dependent manner (Gruhlke et al., 2016) resonating with a preclinical study in mice reported in 1960 (DiPaolo and Carruthers, 1960), thus making it a good candidate for testing in patients with helminth-induced liver and bile duct cancers.

## Conclusion

The chain of molecular and immunological events occurring between infection and neoplasia in humans is challenging to study. Nevertheless, biomarkers obtained via clinical and translational studies (the role of METTL13/FEAT in infection-driven neoplasia is a good example), as well as ongoing drug trials provide an avenue to examine fluctuations which reflect different stages of the immunopathological process. A combination of measurement of soluble biological mediators in blood, immunohistological methods, next-generation sequencing and existing clinical knowledge will be able to identify and design personalized therapies. Furthermore, constituents of the study cohorts in question, as inclusion of uninfected household contacts, unrelated healthy donors and geographical distribution play a remarkably important role to more accurately decipher the tenets of protective vs. pathological immune and genetic biomarkers which may have theranostic value.

## Author Contributions

MR, JL, AZ, and MM initiated, conceptualized, and wrote the first draft of the manuscript. All authors contributed with suggestions, revisions, and interpretation of different aspects in immune-interventions.

## Conflict of Interest

The authors declare that the research was conducted in the absence of any commercial or financial relationships that could be construed as a potential conflict of interest.
